# Clinical predictors of prognosis in stroke patients after endovascular therapy

**DOI:** 10.1038/s41598-024-51356-5

**Published:** 2024-01-05

**Authors:** Yugang Wang, Xingyun Yuan, Yonggang Kang, Liping Yu, Wanhong Chen, Gang Fan

**Affiliations:** Department of Neurology, The First People’s Hospital of Xian Yang City, Xian Yang, Sha’anxi China

**Keywords:** Neuroscience, Neurology

## Abstract

Endovascular therapy (EVT) is effective in the treatment of large vascular occlusive stroke. However, many factors are associated with the outcomes of acute ischemic stroke (AIS) after EVT. This study aimed to identify the main factors related to the prognosis of AIS patients after EVT. We analyzed the clinical data of AIS patients in the neurology department of our medical center from June 2017 to August 2021 following treatment with EVT. The data included the patients’ blood pressure upon admission, blood glucose concentration, National Institutes of Health Stroke Scale (NIHSS) score, 90-day modified Rankin scale (mRs) score follow-up data, and time from LKN to the successful groin puncture (GP). A good outcome was defined as a 90-day mRs score of 0–2, and a poor outcome was defined as a 90-day mRs score of 3–6. A total of 144 patients were included in the study. Admission, smoking, and LKN-to-GP time, NIHSS score of 6–12 was found to be relevant to the prognosis. The results of multivariate analysis showed that prognosis was significantly influenced by baseline NIHSS (odds ratio = 3.02; 95% confidence interval, 2.878–4.252; *P* = 0.001), LKN-to-GP time (odds ratio = 2.17; 95% confidence interval, 1.341–2.625; *P* = 0.003), and time stratification (6–12 h) (odds ratio = 4.22; 95% confidence interval, 2.519–5.561; *P* = 0.001). Our study indicated that smoking, baseline NIHSS score, and LKN-to-GP time were the risk factors for a poor outcome in stroke patients following an EVT. Quitting smoking and shortening LKN time to GP should improve the outcome of AIS after EVT.

## Introduction

Stroke is one of the main causes of death among the elderly people. In addition, the mortality and disability rates after stroke are 115/100,000 (95% confidence interval, 96–133) and 2.77% (Estonia) to − 0.23% (Romania), respectively, which results in serious consequences for the patient’s family and reduces the patient’s quality of life^1^. Endovascular therapy (EVT) is highly efficacious for acute ischemic stroke (AIS) due to large-vessel occlusion when performed within 24 h after the onset in selected cases^2^. Recent advances in AIS, which is caused by large-vessel occlusion and treated with EVT, have reduced the severity of disability, morbidity, and mortality rates of stroke^3^. In our medical center, the ratio of patients with AIS treated with EVT was 52% (154/302). The mortality of AIS patients treated with EVT was approximately 10% (16/154), which was lower than that of AIS patients who only received medical treatment (12.8%, 19/148, *P* < 0.05). About 6–7% of AIS patients were eligible to receive EVT^4^. With the increasing number of AIS patients who receive EVT, the benefits of EVT are also increasing. A previous study showed that clinical outcomes of EVT patients with failed reperfusion did not differ significantly from the outcomes of patients treated with best medical management; moreover, not all patients with large-vessel cerebral infarction can benefit from EVT^5^. In this study, we retrospectively analyzed the data of patients from our center who had undergone EVT—which is now indicated in patients within 24 h from their last known normal (LKN), provided that they meet specific clinical and imaging criteria —We want to analyze the factors associated with prognosis. This study aimed to identify the main factors that promote poor prognosis in AIS patients after EVT.

## Materials and methods

### Study population and parameter definitions

**Figure 1:**
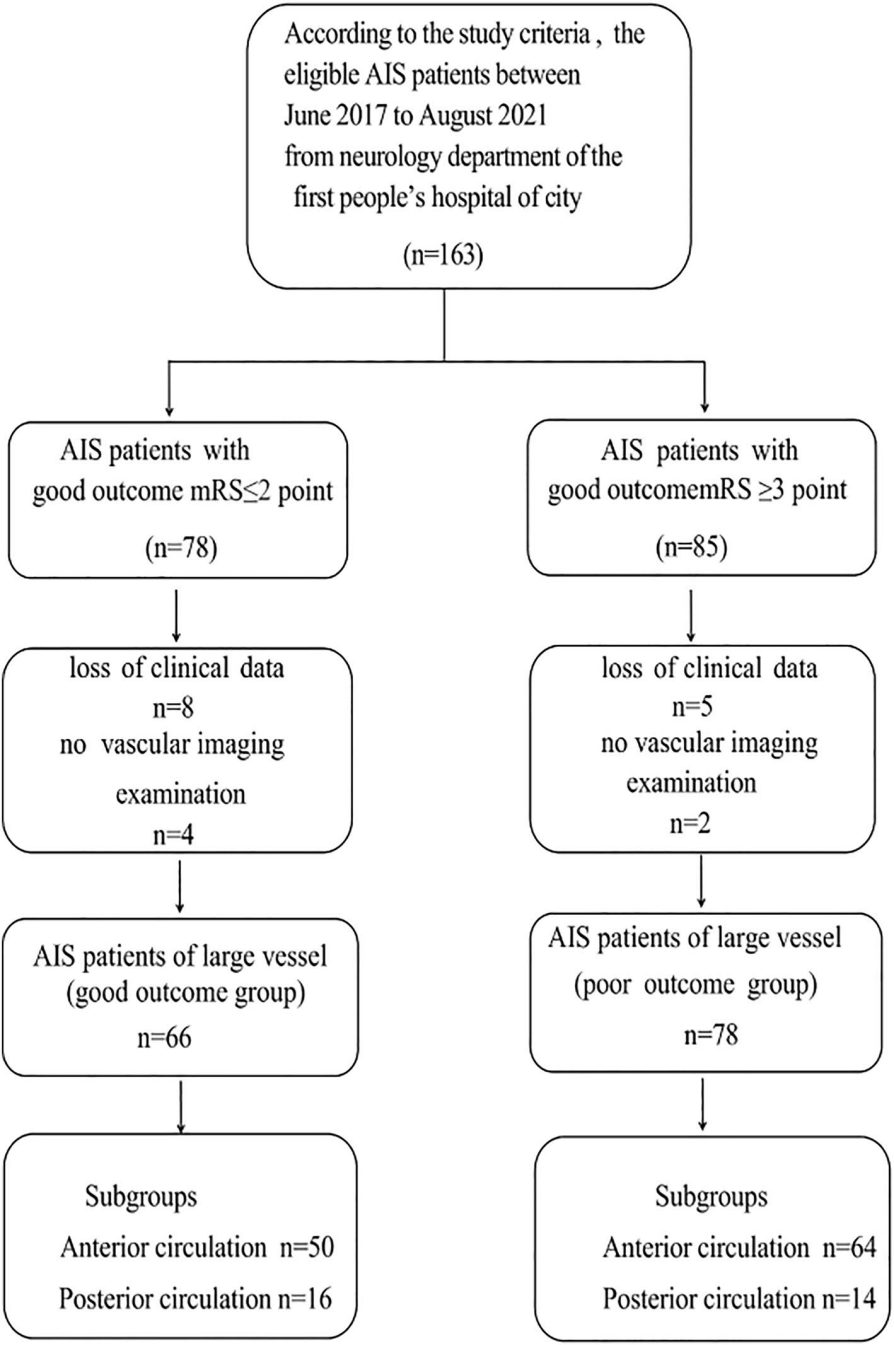
The flowchart of recruitment and selection process.

All 163 patients treated with EVT at our academic stroke center from June 2017 to August 2021 were recruited into the study. 19 patients were eliminated for the lack of clinical and imaging examination data, and 144 stroke patients (including anterior and posterior circulation large-artery occlusions) were finally included (Fig. 1). The time of disease onset was within 24 h. The patients met specific clinical and imaging criteria^6^. Each patient was followed up after 90 days. According to the time from stroke onset to visit time (0–3, 3–6, 6–12, > 12 h, unknown), the patients were stratified into five groups (Table 1). The patients underwent several examinations, including digital subtraction angiography (DSA), noncontrast computed tomography (CT), CT angiography (CTA), and CT perfusion. Those who went to hospital late underwent noncontrast CT^7,8^ and magnetic resonance angiography (MRA). The image checking, selection, and evaluation were conducted by two experienced radiologists.

**Table 1:** The clinical baseline data of 144 stroke patients.

Item	Overall (n = 144)	Good Outcome (n = 66)	Poor Outcome (n = 78)	*P* value
Age	65.41 ± 11.45(yrs)	67.72 ± 10.42(yrs)	63.65 ± 11.94(yrs)	0.19
Gender (male/female)	80/64	38/28	42/36	0.23
Hypertension	61/144	23/66	38/78	0.15
Anterior Circulation	114	50/114	64/114	0.31
Posterior Circulation	30	16/30	14/30	0.18
Dyslipidemia	8/144	3/66	5/78	0.20
Atrial Fibrillation	60/144	27/66	33/78	0.25
Diabetes mellitus	13/144	5/66	8/78	0.19
Glucose(mmonl/L)	9.2 ± 2.31	8.3 ± 2.32	10.3 ± 3.04	0.11
LDL (mmonl/L)	4.2 ± 1.17	3.8 ± 1.43	4.5 ± 2.56	0.17
INR	1.1 ± 0.45	0.9 ± 0.46	1.2 ± 0.87	0.15
Systolic blood pressure(mmHg)	161 ± 35	167 ± 34	155 ± 52	0.23

Good Outcome: modified Rankin scale (mRs)≦2, Poor Outcome: mRS≧3, *INR* international normalized ratio; *LDL* low density lipoprotei.

The inclusion criteria were as follows: (1) patients were at least 18 years old; (2) the diagnosis of AIS due to large-vessel obstruction (LVO) was based on clinical presentation and imaging examinations, including MRA, CTA, or conventional DSA. In CTA, MRA, and DSA, patients with AIS presented with blockage of large cerebral blood vessels; (3) patients were admitted to neurology wards; (4) patients were conscious, cooperative, and able to provide all necessary information. Patients were excluded if they were diagnosed without imaging examinations; they had venous vascular disease, head trauma, acute intracranial infection, renal or hepatic failure, acute myocardial infarction, chronic heart failure, or hematological malignancy; or they were unwilling to cooperate or unable to provide reliable information.

### Data collection

The collected data included the patients’ age, gender, LKN-to-GP time, neurological deficit (National Institutes of Health Stroke Scale [NIHSS]), laboratory test results, common risk factors for stroke (hypertension, diabetes mellitus, atrial fibrillation, and smoking history), symptomatic intracerebral hemorrhage (sICH) within 24 h after EVT, and time stratification (time since stroke onset). NIHSS was obtained by depending on calculating by the investigators of this study, and laboratory test data collected before undergoing EVT. While a number of scores could be used in the evaluation of stroke severity, the mRs scale—which represents the severity of functional impairment—is generally used in stroke research; mRS score takes values of 1, 2, 3, 4, 5, or 6, where a higher score indicates more serious impairment. A favorable prognosis was defined as an mRs score of 0–2, and a score ≥ 3 was defined as a poor outcome at 90 days after AIS.

### Statistical analysis

The data were analyzed using SPSS software (IBM SPSS Statistics for Windows, version 22.0, New York, U.S.A.). Continuous variables were shown as mean ± SD, or the median (interquartile range [IQR]). Student’s *t*-test was carried out on the data that conformed to a normal distribution (Shapiro–Wilk test, *P* ≥ 0.05). If the data were collected as rates, the χ^2^ test was used. Multivariate binary logistic regression analysis for the predictors of favorable clinical prognosis was performed for all variables that were significant at the *P* < 0.05 level in the univariate analyses. Discrete numeric variables were depicted by medians (IQR). Age, gender, and other factors were adjusted for in the multivariate binary logistic regression analysis. A value of *P* < 0.05 was considered statistically significant.

### Ethical approval

This study was approved by the Ethical Committee of First People’s Hospital of Xian Yang city in Sha’anXi province. All patients signed an informed consent approved by the review board. Research involving human participants, human material, or human data, must have been performed in accordance with the Declaration of Helsinki. All procedures carried out in studies involving human participants were in accordance with the ethical standards of the institutional and national research committee. The ethical approval ID of the study (No:20230201).

## Results

**Figure 2:**
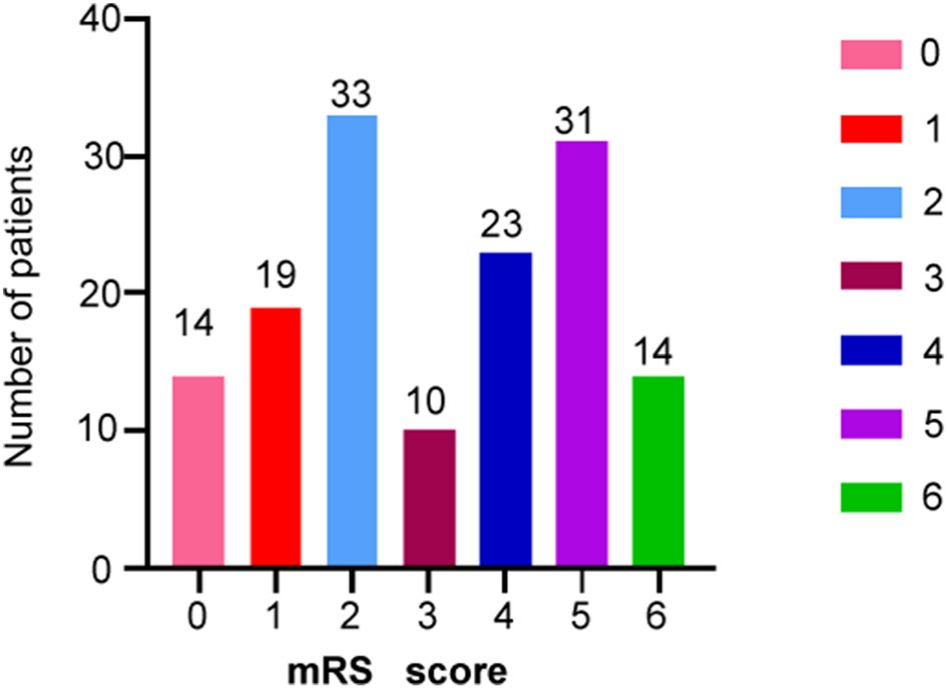
The mRS score information of 144 stroke patients (0–2 means a good outcome, and ≥ 3 means a poor outcome).

A total of 144 patients were analyzed in our study. 66 patients had mRs scores of 0–2, and 78 patients had mRs scores ≥ 3 (Fig. 2). The mean age was 64.4 ± 12.6 years, namely 67.7 ± 10.4 years in the favorable-prognosis group and 63.6 ± 11.9 years in the poor-prognosis group (*P* = 0.19). In our study, the anterior/posterior circulation ratio of the two groups was not significantly different (*P* > 0.05, Table 1). The admission NIHSS scores and other baseline clinical data are displayed in Table 1. There were no significant intergroup differences in the following factors: hypertension history (23/66 vs. 38/78, *P* = 0.15), dyslipidemia (3/66 vs. 5/78, *P* = 0.20), diabetes mellitus (5/66 vs. 8/78, *P* = 0.19), atrial fibrillation (27/66 vs. 33/78, *P* = 0.25), glucose concentration (8.3 ± 2.32 vs. 10.3 ± 3.04, *P* = 0.11), low-density lipoprotein–cholesterol (LDL) concentration (3.8 ± 1.43 vs. 4.5 ± 2.56, *P* = 0.17), International Normalized Ratio (INR: 1.0 ± 0.46 vs. 1.2 ± 0.87, *P* = 0.15), and systolic blood pressure (mm Hg) (167 ± 34 vs. 155 ± 52, *P* = 0.23) (Table 1). Smokers had a poorer outcome than nonsmokers (19/66 vs. 36/78, *P* = 0.005). The baseline NIHSS score was lower in the patients with a favorable prognosis than in those with a poor prognosis (10.2 vs. 14.23, *P* = 0.019). The LKN-to-GP time was shorter in the favorable-outcome group than in the poor-outcome group (350 ± 71 vs. 570 ± 92 min, *P* = 0.003). According to the stratification of the onset time, the number of patients with a poor prognosis was higher than other groups (6–12 h, stratification of onset to visit time). The prognosis of stroke patients with sICH (PH2) after stroke was often poor (Table 2).

**Table 2:** The clinical data of 144 stroke patients after endovascular treatment.

Item	Overall (n = 144)	Good Outcome (n = 66)	Poor Outcome (n = 78)	*P* value
Smoking	55/144	19/66	36/78	0.005
Baseline NIHSS	12.56 [10.04, 16.32]	10.2 [7.95, 13.26]	14.23 [10.81, 18.21]	0.019
LKN to grion puncture (mins)	406.13 [296.47,499.53]	350.34 [241.73,427.41]	570.19 [404.83,724.14]	0.003
Time stratification (h)				
0–3	15/144	5	10	0.155
3–6	54/144	22	32	0.120
6–12	36/144	24	12	0.005
> 12	10/144	4	6	0.232
Unknown	29/144	11	18	0.271
NIHSS stratification				
< 10	39/144	25	14	0.002
10–20	78/144	34	43	0.115
> 20	27/144	5	22	0.002
Safety (sICH)				
PH-1	5/144	3	2	0.112
PH-2	9/144	2	7	0.009

*INR* international normalized ratio, *LDL* low density lipoprotein, *LKN* last known normal, *Time stratification* Stratification of onset to visit time; Modified Thrombolysis in Cerebral Infarction, *NIHSS* National Institutes of Health Stroke Scale, *sICH* symptomatic intracranial hemorrhage, and *PH* parenchymal hematoma.

**Figure 3:**
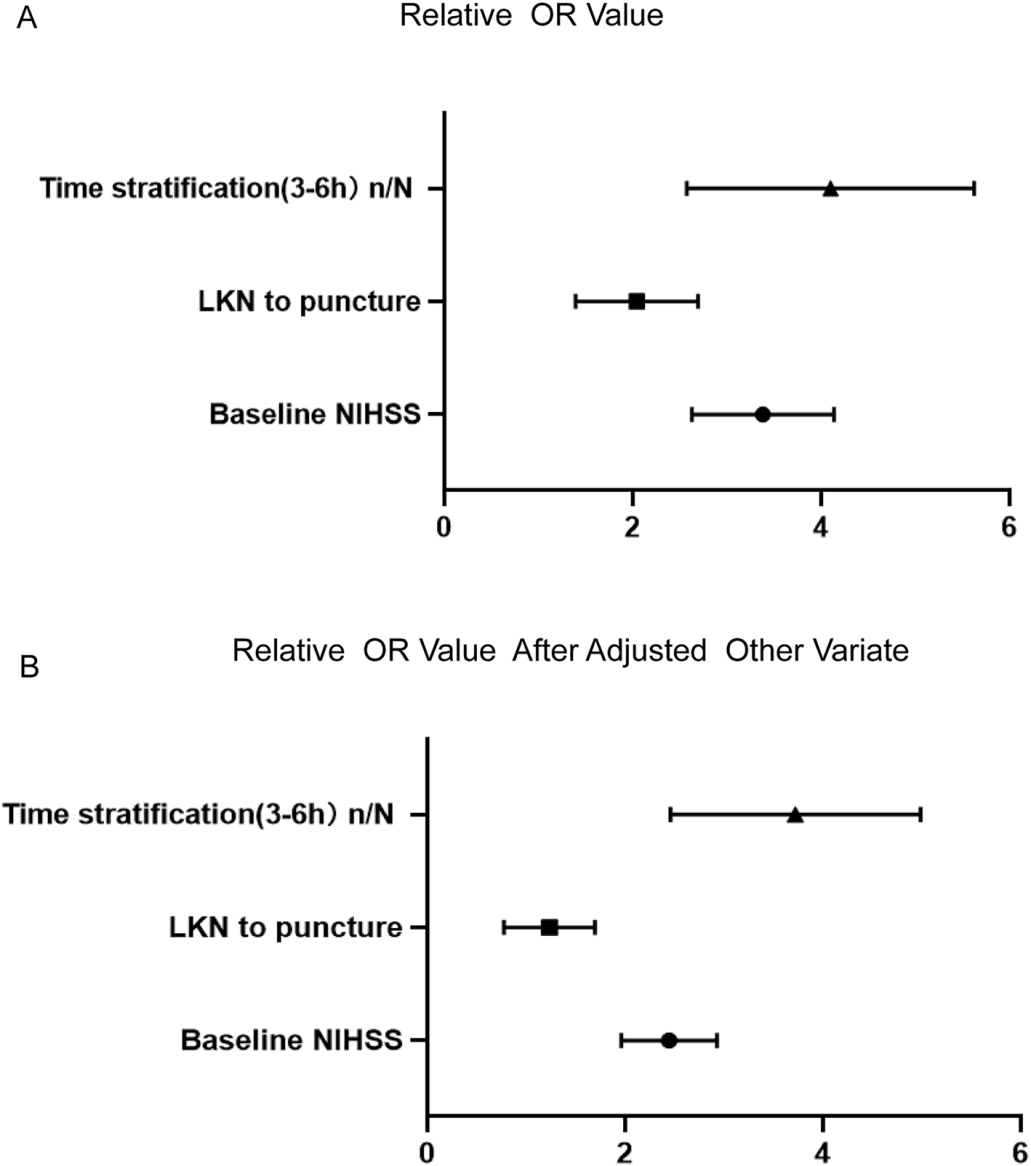
The odds Ratio (OR) of smoking, baseline NIHSS and LKN time to puncture as in the poor outcome group were significantly higher than good outcome group (**A**, **B**).

Baseline NIHSS, LKN-to-GP time and time stratification (6–12 h), age, gender, arterial hypertension, diabetes mellitus, smoking, random blood glucose, and LDL were included into the regression model. Baseline NIHSS, LKN-to-GP time, and time stratification (6–12 h) were closely associated with the outcome. After adjusting for the covariates, baseline NIHSS (OR = 2.57, *P* = 0.023), LKN-to-GP time (OR = 1.17, *P* = 0.035), and time stratification (6–12 h) (OR = 3.819, *P* = 0.015) were still closely associated with the outcome (Fig. 3). The data are displayed in Table 3.

**Table 3:** Multivariable binary logistic regression analyses outcome according to status in stroke patients treated with endovascular treatment.

Items	B	Wald	*P* value Unadjusted [OR, 95% CI]	*P* value Adjusted [OR, 95% CI]
Baseline NIHSS	−1.45	1.533	0.001 3.02 (2.878–4.252)	0.023 2.57 (1.912–2.853)
LKN to puncture (minutes)	1.307	2.621	0.003 2.17 (1.341–2.625)	0.035 1.17 (0.812–1.723)
Time stratification(3–6 h)n/N	2.508	23.01	0.001 4.22 (2.519–5.561)	0.015 3.819 (2.412–4.937)

*NIHSS* National Institutes of Health Stroke Scale, *LKN* last known normal, *OR* Odds ratio, *CI* Confidence interval.

## Discussion

EVT is effective in the treatment of large vascular stroke. However, the prognosis of stroke is affected by many factors^9^. A previous study has shown that EVT in patients with vertebrobasilar artery occlusion may improve the long-term prognosis^10^, and a higher level of cerebral edema is associated with a poor prognosis in AIS patients eligible for EVT^11^. A previous study has indicated that near complete or complete revascularization (TICI 2c/3) is associated with higher rates of functional outcomes after EVT^12^. In our research, the baseline NIHSS score, LKN-to-puncture time, smoking, time stratification, and PH2 showed a clear correlation with the outcome in the patients with AIS after receiving EVT. Stroke is prevalent worldwide and has a high morbidity rate. Many scales are used to measure the severity and outcome of stroke. The NIHSS is a quantifiable scale that is used to evaluate the severity of stroke^13^. The patients who had a low baseline NIHSS score often had more favorable clinical outcomes than the patients with a high baseline NIHSS score. A previous study, which analyzed 484 stroke patients, showed that the recanalization in patients with an NIHSS score of 5 (IQR, 4–7) (thrombolysis in cerebral infarction, TICI 2b–3) was achieved in 26 (78.7%) patients^14^, and the patients all had favorable clinical outcomes. A low baseline NIHSS score was independently predictive of a favorable outcome in patients with posterior-circulation (odds ratio [OR] 1.547; 95% confidence interval [CI], 1.232–1.941) and anterior-circulation (OR, 1.279; 95% CI, 1.188–1.376) stroke. The baseline NIHSS score for a favorable functional outcome in anterior-circulation stroke was 4 (IQR, 3–7), compared with 3 (IQR, 1–5) for posterior-circulation stroke^15^.

Time was related to the clinical outcomes. A previous study showed that the outcomes could be enhanced by reducing the door-to-GP time^16^. Our study suggests that any effort to improve the time from LKN to GP time will result in a positive outcome. A study by Sun et al. showed that when the logistic regression model was adjusted for age, NIHSS, hypertension, diabetes mellitus, reperfusion status, and symptomatic hemorrhage, the preprocedural time frame from LKN to GP was directly associated with 90-day positive outcomes (OR, 0.996; 95% CI, 0.993–0.998; *P* < 0.001)^17^. Another study also showed that shorter LKN-to-GP time improved the outcome, particularly in those with good ASPECTS (CT stroke score system) presenting within 6 h. Strategies to decrease reperfusion times should be investigated, particularly early on and in patients with good ASPECTS^18^. Our study is consistent with the previously reported studies.

Smoking is a major risk factor for many diseases, including stroke, especially among young and middle-aged stroke patients. Our study also showed that the percentage of smokers among the patients with poor outcomes was higher than that among the patients with favorable outcomes^19^. A previous study showed a strong dose–response relationship between cigarettes smoked daily and ischemic stroke among young men. The study was a population-based case–control study of risk factors for ischemic stroke in men aged 15–49 years. The OR for the current-smoking group compared with the never-smoking group was 1.88. Although complete smoking cessation is the goal, even smoking fewer cigarettes may reduce the risk of ischemic stroke in young men^20^. The relationship between smoking and stroke is controversial. According to some studies, smoking in stroke patients could be beneficial after EVT. However, these data should not be misunderstood as a benefit of smoking. Another study reported that smoking was not associated with a good functional prognosis (mRS ≤ 2) at 3 months in AIS patients who were treated with intravenous thrombolysis^21^. Due to the controversial effects of cigarette smoking on cardiovascular health, smoking cessation is still recommended for stroke prevention^22^.

The sooner a stroke patient is treated, the better the prognosis. As shown in a multicenter randomized clinical trial of endovascular treatment for AIS in the Netherlands, patients treated for an occlusion of the intracerebral internal carotid artery or the middle cerebral artery within a 6-h window from the LKN time benefit from EVT^23^. A previous study showed that EVT could benefit patients and provided evidence of reversible cerebral ischemia in the time window of 6–24 h. The previous study findings suggested that in AIS patients, EVT should be carried out based on the mode of presentation or the time of presentation within the 6–24-h time window^13^.

sICH is the most serious complication after IVT and EVT. Using a combination of the predictors that were available both before and at the end of bridging therapy and direct thrombectomy may provide indications for the early identification of patients who are candidates for a more intensive postprocedural management. In those patients who are at a high risk of sICH, the monitoring and treatment of hypertension and hyperglycemia should be intensified. Patients who are at a low risk of sICH should be transferred back to the referring hospital quickly in a sedative state^24^. A previous study found no difference in the rates of sICH between primarily utilized stent retriever devices and non-stent retriever devices, and the incidence of sICH in care-dependent stroke patients was similar to that in care-independent patients^25^. Our data suggested that the incidence of sICH was higher than previously reported (10% vs. 6%)^26^, and the type PH2 occurred more often in the poor-outcome group than in the favorable-outcome group.

There are two biases or limitations in the current work. First, many factors influence the stroke outcomes, and our study did not consider all factors related to stroke. Second, the stroke outcomes also correlate with early rehabilitation, but our study did not include the early rehabilitation factor.

## Conclusions

Smoking, baseline NIHSS score, and LKN-to-GP time were the risk factors for a poor outcome of stroke patients following an EVT. Quitting smoking and shortening LKN-to-GP time should improve the outcome of AIS after EVT.

## Data Availability

Requests for access to the dataset from qualified researchers trained in human subject confidentiality protocols may be sent to Department of Neurology, first people’s hospital of Xianyan city. The contacting person is the corresponding author and E-mail is concocon@163.com.
