# Author Correction: Clinical predictors of prognosis in stroke patients after endovascular therapy

**DOI:** 10.1038/s41598-024-59017-3

**Published:** 2024-04-09

**Authors:** Yugang Wang, Xingyun Yuan, Yonggang Kang, Liping Yu, Wanhong Chen, Gang Fan

**Affiliations:** Department of Neurology, The First People’s Hospital of Xian Yang City, Xian Yang, Sha’anxi China

Correction to: *Scientific Reports* 10.1038/s41598-024-51356-5, published online 05 January 2024

The original version of this Article contained an error in Figure 2 labelling, where number of patients was incorrect.

The original Figure 2 and accompanying legend appear below.

**Figure 2:**
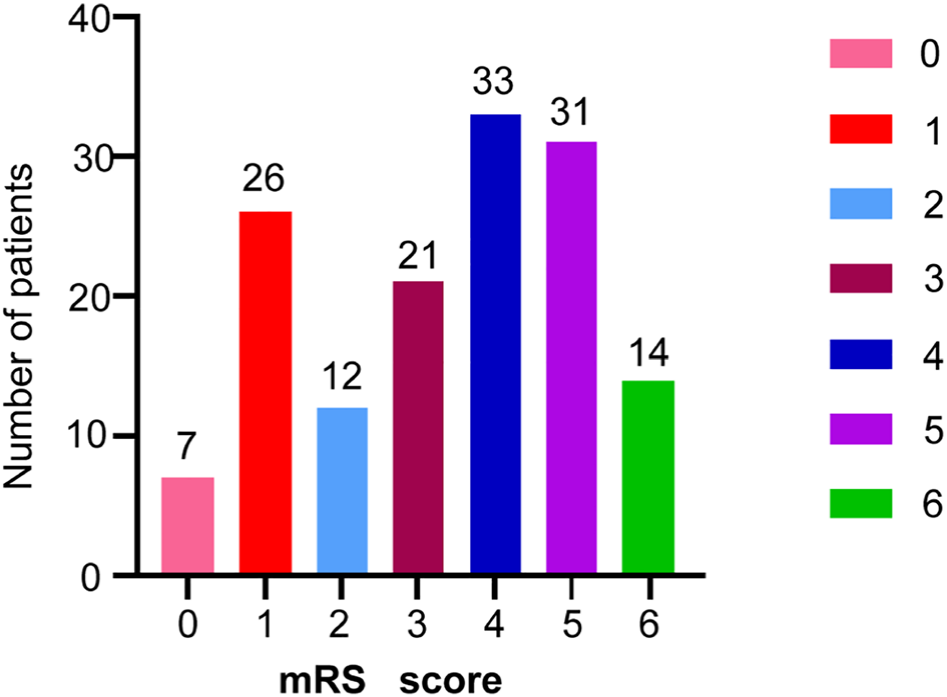
The mRS score information of 144 stroke patients (0–2 means a good outcome, and ≥ 3 means a poor outcome).

The original Article has been corrected.

